# The Epidemiology of Tobacco Use among Khat Users: A Systematic Review

**DOI:** 10.1155/2015/313692

**Published:** 2015-07-26

**Authors:** Saba Kassim, Mohammed Jawad, Ray Croucher, Elie A. Akl

**Affiliations:** ^1^Barts and The London School of Medicine and Dentistry and Institute of Dentistry, Queen Mary University of London, London E1 2AT, UK; ^2^Department of Primary Care and Public Health, Imperial College London, London W6 8RP, UK; ^3^Academic Unit of Primary Care and Population Sciences, University of Southampton, Southampton SO16 6YD, UK; ^4^Department of Internal Medicine, American University of Beirut, Beirut, Lebanon; ^5^Department of Clinical Epidemiology and Biostatistics, McMaster University, Hamilton, ON, Canada

## Abstract

Khat, an “amphetamine-like green leaf,” may influence the consumption of tobacco. This study reviews the epidemiology of tobacco use among khat users. Electronic database searches using appropriate keywords/terms were conducted to identify observational studies of khat use. Assessment of quality and risk of bias of all included studies was conducted, and the results were synthesised descriptively. Nine eligible cross-sectional studies were identified. All assessed self-reported tobacco among khat users and were carried out in Africa and the Middle East. Eight reported cigarettes and one reported waterpipes as the mode of use. Methods of tobacco use prevalence assessment varied. Prevalence of “current” tobacco use among students and university teachers ranged from 29 to 37%; “lifetime” tobacco use in university teachers was 58% and “undefined” tobacco use in nonspecific adults and students ranged from 17 to 78%. Daily tobacco use among adults was reported as 17% whilst simultaneous tobacco and khat use was reported as between 14 and 30% in students. In conclusion, tobacco prevalence among khat users appears significant. Findings should be interpreted cautiously due to self-reported tobacco use, diversity in questions assessing tobacco use, and type of tobacco consumption. Future research should address the methodological shortcomings identified in this review before appropriate policy interventions can be developed.

## 1. Introduction

Tobacco smoking is a significant cause of preventable death and ill health worldwide [1]. Based on current trends, 80% of tobacco-related mortality is predicted to occur in low and middle income countries [2]. Reduction/control of tobacco use in these countries is one of the Millennium Development Goals [3]. Ethnicity/culture alongside other factors (e.g., socioeconomic status) contributes to the uptake of tobacco [4–6], although determinants of tobacco use are complex [7].

The khat leaf is an “amphetamine-like” stimulant [8] that is socioculturally embedded and widely practiced in certain areas of Africa and the Arabian Peninsula [9] and in the diaspora communities from these regions [10, 11]. Khat is an acceptable and habitual practice for these populations, specifically among Muslims [12, 13]. Khat may also be used by students to prevent fatigue when studying [14, 15]. Importantly, in countries where khat is endemic (e.g., Yemen) or among their diaspora, khat is often used within the family [12, 16, 17]. For males, khat is often initiated during early adolescence or even before [15, 18], and for females it may be initiated in late adolescence [15] or after marriage [11, 17].

Khat is often chewed; users may place tender khat leaves in the buccal sulcus and chew for a while and then store the bolus in the pouch of the cheek, often in the left side of the mouth [19] to allow the juice to be systemically absorbed through the oral mucosa [20]. Factors that contribute to the spread of khat use in homeland and diasporas include the deviation from cultural norms of use (e.g., using khat at night) [9, 21]. In addition to this, khat is affordable, accessible, and available throughout the year and in multiple settings [22, 23]. Policies to curb widespread khat agriculture [22, 24] and reduce the importance of khat as a cash crop, as it is in Kenya, are absent [25]. Frequent khat use is associated with negative general, oral, and mental health outcomes [26]. Khat use has become a national and international public health concern, with many countries such as the United Kingdom banning its use [27, 28]. Such an intervention awaits evaluation. Anecdotal evidence suggests powdered and dried leaves have emerged as a replacement to the khat leaf in the UK [29].

Khat is often used in groups and is associated with using other substances such as alcohol [30, 31] and commonly tobacco [32, 33]. Information about the role tobacco and khat play in each other's initiation is scarce [34], and evidence suggests some khat users may only use tobacco during sessions of khat [10, 32, 33, 35, 36]. Dual khat and tobacco users may increase their tobacco consumption during khat sessions [10, 11, 32] and one study showed that regular tobacco smokers were ex-khat users [37]. Khat use may serve as a “gateway” to tobacco use: 12–30% of khat users in the diaspora and homeland report initiation or use of tobacco only when using khat (simultaneous tobacco and khat users (STKU)) [10, 36]. Daily cigarette smokers and STKU report that smoking tobacco enhances the impacts of khat [32]. Also, those who are both regular (daily) tobacco user khat users and STKU reported smoking tobacco more during the first hours of khat use [32] and daily cigarette smoker khat users continued smoking after finishing khat use [32]. Finally, the cooccurring of khat and tobacco smoking dependence is growing [10, 32]. Therefore, there is a possibility that khat use interacts with tobacco use, which may undermine tobacco cessation programs.

The influence of khat use on aspects of tobacco use has not been assessed systematically. We seek in this review to inform the scientific debate about the neglected public health issue that khat use is often associated with tobacco. Our primary aim is to systematically identify, appraise, synthesise, and summarise the best available evidence on the epidemiological association between khat use as the exposure and tobacco use (prevalence, pattern, and mode of tobacco) as the outcome. The secondary aim is to explore factors associated with concurrent tobacco and khat use and the level and methods used for measuring tobacco dependence amongst khat users. The review question is as follows: What is the best available evidence on the epidemiology of tobacco among khat users?

## 2. Materials and Methods

A protocol for this review has been published in Prospero [38].

### 2.1. Eligibility Criteria

We used the following inclusion criteria for our systematic review:

- Original quantitative (cross-sectional and cohort) studies.
- Studies detailing tobacco epidemiology among khat users.
- Any time frame or population group.

We used the following exclusion criteria:

- Case-control studies, case reports, case series, clinical trials, reviews, and experimental laboratory studies (prevalence cannot be estimated).
- Studies using convenience and purposive sampling (prone to selection bias).
- Studies including tobacco user khat users for specific population, for example, with mental health conditions (prone to confounding).
- Duplicate studies.

### 2.2. Search Strategy

In November 2014 we searched the following electronic databases: MEDLINE (1950–present), Embase (1980–present), PsycINFO (1806–present), and ISI Web of Science. Search terms were “catha,” “miraa,” “qat,” “khat,” and “kath.” These were based on the peer-reviewed literature and the expertise of the research team in the field. We did not combine khat keywords/terms with tobacco keywords/terms to allow pooling all of the available literature of khat. Only full texts written in English or Arabic were considered. We screened the bibliography of review articles for relevant citations. Finally, we created EndNote libraries (software package Endnote XIII) for each database search, merged them, and removed duplicates.

### 2.3. Selection Process

Based on the eligibility criteria, two reviewers (S. Kassim and M. Jawad) independently screened the title and abstract of available citations to identify potentially eligible studies. We retrieved full texts of studies considered potentially eligible by at least one reviewer. The same two reviewers then independently screened full texts using a standardised and pilot-tested screening form, resolving disagreements with the help of a third reviewer (E. A. Akl).

### 2.4. Data Abstraction and Analysis

Two reviewers (S. Kassim and M. Jawad) independently abstracted data from each eligible study using a standardised and pilot-tested data abstraction form, again resolving disagreements with the help of a third reviewer (E. A. Akl). Quality assessment was based on a previous systematic review for observational studies [39]. For all included studies we abstracted data on the methodology (sampling frame, sampling method, recruitment method, and administration method), methodological quality (presence of a sample size calculation, sampling type, validity of tool, presence of pilot testing, and response rate), population and setting (population, country, setting, number of subjects sampled, number of subjects participated, and number of subjects' data items analysed), and prevalence data (prevalence and pattern of khat use and prevalence, pattern, and mode of tobacco use among people who use khat, including biochemical verification). We contacted authors for additional information if not available in the published paper. Other information abstracted included associated factors with dual khat and tobacco use and the levels and methods used for measuring tobacco dependence amongst khat users. Data were analysed descriptively and formulated into a quantitative narrative synthesis. Results were expressed as percentages for the prevalence and frequency with percentages for mode of tobacco delivery and pattern of use.

## 3. Results

### 3.1. Description of Included Studies

**Figure 1:**
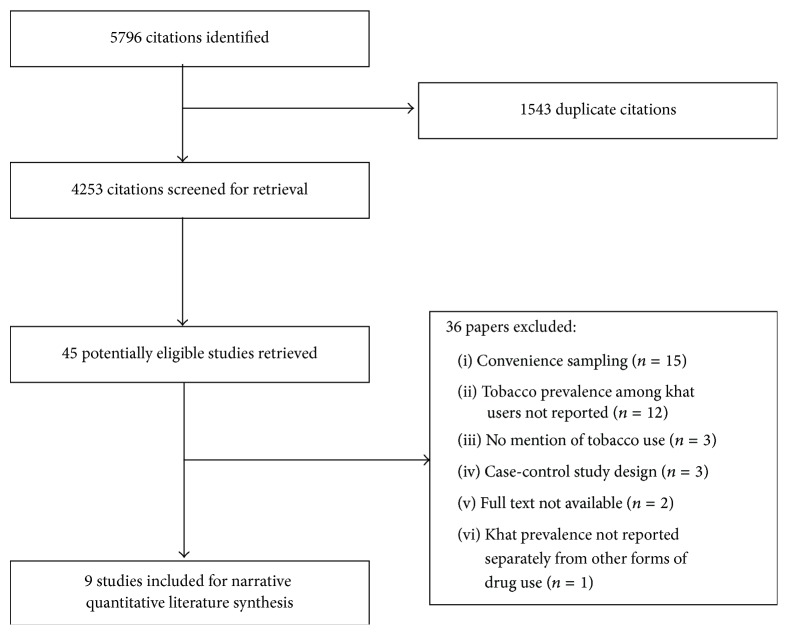
Study flow diagram.

Figure 1 presents the study flow. All studies were identified through electronic searches only. Of 45 considered studies, we excluded 36 studies.

Table 1 provides the full details of the nine included studies, all of which were cross-sectional. The target populations in the nine identified studies were adults (*n* = 4), university students (*n* = 2), and high school and/or college students (*n* = 3).

Studies varied in the way they measured khat prevalence. Some studies opted for measures of regularity, such as daily or weekly use (*n* = 5) [14, 21, 40–42], while others opted for current (*n* = 4) [15, 41, 42, 44] or ever occurring use (*n* = 3) [15, 36, 44]. One study measured khat prevalence as those who used khat for greater than three years [43].

Studies also varied in the way they measured tobacco prevalence among khat users. Most studies opted for current (*n* = 2) [36, 44] or ever occurring use (*n* = 3) [14, 36, 44] whereas others opted for measures of intensity, such as mild or heavy (*n* = 1) [41], or simply the number of cigarette smoked as a measure of prevalence [43]. Over half of studies did not specify the measure of tobacco use (*n* = 5). Eight studies reported cigarettes and one study reported waterpipe [36] as mode of tobacco use. Finally, none of these studies measured level of tobacco dependence among khat users.

### 3.2. Methodological Quality of Included Studies

Three studies included sample size calculations [15, 21, 42]. The instruments used to measure khat prevalence were as follows: previously reported validated tools (*n* = 3) [15, 21, 42], a validated self-developed tool (*n* = 1) [41], and an unvalidated self-developed tool (*n* = 1) [40] and four studies did not report the instrument used (*n* = 4) [14, 36, 43, 44]. Six studies reported pilot testing of the measurement instrument [15, 21, 36, 41, 42, 44]. Seven studies reported a response rate which varied from 70.4% to 96% [15, 21, 36, 40–42, 44] whilst in two studies this was not reported [14, 43].

We used the tool proposed by Siegfried et al. [39] to assess methodological quality. For external validity (representativeness of the sample), seven studies reported representative sample (probability sampling) of the targeted population and two studies reported a broad sample (the whole population was included in the study) [40, 44]. With respect to internal validity, tobacco and khat use was self-reported and any performance bias such as the blindness of the assessor to tobacco and khat use status was not reported. Prevalence estimates were not provided with confidence intervals and there were wide variations in the time frames used for the estimate of prevalence. Adjustments for confounding factors for tobacco and khat use were only reported by one study conducted among doctors in Yemen, which explored the association between cigarette smoking among khat users and sociodemographic factors [40]. Table 1 provides detailed description of the characteristics of included studies.

### 3.3. Epidemiology of Tobacco Use among Khat Users

#### 3.3.1. Prevalence of Tobacco among Khat Users

*Adults*. Three studies measured tobacco prevalence among khat users in Ethiopia. In one study of 10,468 respondents, 8.7% were daily khat users, 1.8% were mild (smoked 1–3 daily) cigarette smokers, 1.3% were moderate (smoked 4–9 daily) cigarette smokers, and 1.3% were heavy (smoked > 9 daily) cigarette smokers. Among the 8.7% daily khat users, 5.0% were mild cigarette smokers, 5.2% were moderate cigarette smokers, and 6.5% were heavy cigarette smokers [41]. In a second study [44] among mainly male university instructors, 32.6% were ever khat users, 21.0% were current (past 30 days) khat users, 28.2% were ever cigarette smokers, and 13.3% were current (past 30 days) cigarette smokers. Among users who ever used khat, 57.6% were ever cigarette smokers, and, among current khat users, 36.8% were current cigarette smokers.

In one study of 568 doctors in Yemen, 44.0% were khat users (defined as sometimes, frequently, or daily) and 17.6% were cigarette smokers. Among khat users, the prevalence of cigarettes use was 33.9% [40]. Finally, in a study of 1500 Yemenite Jews, 6.8% used khat for greater than three years. Among khat users 68.0% were cigarette smokers and khat users smoked more than nonkhat users (29.5 versus 22.3 cigarettes/day, *p* = 0.03) [43].

*Students*. Two studies measured tobacco prevalence amongst khat using university students in Ethiopia. In one study among 2,230 respondents, 10.7% used khat at least 1-2 times per week and 3.8% were ever cigarette smokers. Of the khat users, 28.7% were current cigarette smokers and 2.4% were former cigarette smokers [14]. In another study of 472 respondents 24.8% were current (daily, weekly, or occasionally) khat users and 13.6% were cigarette smokers. Among current khat users 45.3% were cigarette smokers, 13.7% smoked cigarettes while using khat, and 6.8% continued to smoke cigarettes after completing a session of khat chewing [42].

Among high school students, three studies measured tobacco prevalence among khat users. In a study of 8,965 students in Saudi Arabia, 20.0% were current (past 30 days) khat users and 9.6% were cigarette smokers. 78.4% of current khat users were also cigarette smokers [21]. In a second Saudi Arabian study of 3,923 students, 20.5% were current (past 30 days) khat users and 17.3% were cigarette smokers. Amongst current khat users, 54.3% were cigarette smokers [15]. In the third study of 1,721 students in Ethiopia, 24.2% had ever used khat and 4.2% were current (past 30 days) smokers. Among users who ever used khat, 128/427 (29.9%) used waterpipe when using khat [36].

#### 3.3.2. Factors Associated with Tobacco and Khat Use

A logistic regression model adjusted for age, marital status, residence, and income found that cigarette smoking among khat users was significantly associated with male gender (AOR 3.77, 95% CI 1.10, 12.92) working in governmental and private sectors compared to working in government only (AOR 0.40, 95% CI 0.21, 0.75) and with working greater than 10 years compared to less than 10 years [40].

## 4. Discussion

### 4.1. Key Findings

This review evaluated the epidemiology of tobacco use among khat users. We demonstrated that tobacco prevalence among khat users appears significant. Particularly worrying are high levels of use among high school, college, and university students. The main pattern of tobacco use was daily cigarette smoking, although two studies identified instances of simultaneous tobacco and khat users (STKU). The main mode of tobacco use was cigarettes, which was reported in eight out of nine studies.

### 4.2. What This Study Adds and Confirms

This is the first review to report on tobacco epidemiology among khat users, and it benefits from its systematic methodology. The cooccurrence of tobacco use among khat users may be underpinned by many potential mechanisms which await further exploration in research of better quality. One should consider that khat use often occurs in group sessions in which tobacco use is prevalent [32, 45, 46]; the likelihood of conditioning (use of tobacco with khat) among naïve khat users should be considered as it has been reported elsewhere [47]. In addition to this, khat users reported that tobacco enhances khat effects [32]. Notably, the use of khat and hence associated tobacco among school children and colleagues and university students has been highlighted here in this study. Whilst students use khat to accommodate for their academic commitments and to keep them awake at nights to study [48], one should consider as well the use of khat and tobacco in school children to be multifactorial [21] though the likelihood of the family context in children use is plausible [17].

This review has lent further support to the current literature of social (particularly ethnocultural) determinants of tobacco use [4, 5, 49]. Tobacco use is embedded within the culture of khat and in certain geographic areas, namely, areas of Africa and the Middle East. Tobacco use among khat users also appears to be irrespective of religion, as our review identified both Muslim and Jewish population groups [15, 40, 43]. Furthermore, tobacco use among khat users may be irrespective of level of education or income. Not only was a significant level of tobacco use reported among university teachers [44] and health care providers who used khat [40], but also high income (measured by proxy of working in government and private sector) was associated with dual use [40]. These findings lend further support to the complexity of tobacco use and support the argument that tobacco use is context dependent and has its specific determinants [7]. A number of khat users were identified in this review to be former tobacco smokers and the likelihood of reinitiating tobacco use when using khat is plausible as reported elsewhere [34].

### 4.3. Limitations and Strengths

The limitations of our review include the exclusive inclusion of studies published in English and Arabic and not searching the grey literature. Remaining limitations relate to the shortcomings of included studies. Indeed, these studies assessed only self-reported tobacco use with no biochemical verification (e.g., carbon monoxide). Ascertaining tobacco use biochemically may eliminate the performance bias of the assessor, recall bias, and social desirability bias [39, 50], particularly among female khat users for whom cigarette use is stigmatised [33, 46]. No studies elicited tobacco use with standardised questionnaires (e.g., WHO Global Adult Tobacco Survey) so we could not compare tobacco use in different settings and populations of khat users. In addition, the diversity of the background of khat users and pattern of tobacco use (daily or STKU) should be considered when trying to infer the epidemiology of tobacco use among khat users. Nevertheless, all studies have shown the association between khat use and the epidemiology of tobacco consumption (prevalence, pattern, and mode of use) in different population and setting of khat users.

### 4.4. Future Research and Policy Implications

Researchers measuring the prevalence of tobacco among khat users should ensure the use of validated tobacco questionnaires and include items that identify those that are STKU. Future studies should estimate uses of tobacco among specific group khat users “at risk” (pregnant and diabetic patients) as well as patients with mental health disorders. Importantly, a high prevalence of tobacco use among female khat users has been reported elsewhere [33] and we have reported that tobacco uses among khat users are more likely to be by male [40]. Rigorous mixed methods approaches that address the relationship of khat and tobacco use should explore the determinants of dual use, the perception of tobacco status among those who smoke tobacco only during a khat session, and the levels of dependence among these users; all while appreciating other forms of khat (e.g., powdered and dried leaf versions) may be used.

Currently a lack of knowledge exists about aspects of STKU among certain groups who demonstrate khat use disorders (e.g., daily khat users). These groups are likely to reside in countries where khat is widespread and socioculturally embedded, such as East Africa and the Arabian Peninsula. Whilst the psychosocial and biobehavioural factors of khat use need to be developed and expanded to understand its influences, the concern is mainly related to the indirect impact of khat use on the uptake of tobacco. In all studies, we found that the prevalence of tobacco among khat users was higher than among tobacco users alone in all populations and in different settings. Importantly, the level of tobacco use among students in school or children is worrying; tobacco is addictive and the risk of tobacco dependence increases when smoking begins early [51]. Yet, for example, the khat-endemic Yemen has ratified the WHO FCTC [52] but we only identified one Yemeni study meeting the methodological rigour that addressed the prevalence of tobacco among khat users, and this was among healthcare providers [40]. Population-level behavioural surveillance data to explore tobacco use specifically embedded in khat should be undertaken. This surveillance may guide effective mechanism(s) that involve professional policy makers. As we have previously outlined achievement of the Millennium Development Goals is possible if a main focus becomes the reduction of tobacco use in poor countries [3].

## 5. Conclusions

The prevalence of tobacco use among khat users appears significant, specifically, among high school students, university students, and health care workers in certain African countries and the Middle East. Patterns of use were either daily tobacco use or only using tobacco during khat sessions. The study underscores many knowledge gaps and methodological shortcomings in studies that measure tobacco and khat prevalence. Policy should take into account the current changes in the khat market in the diasporas and the impacts that may contribute to tobacco use. Meanwhile future research should explore the level and nature of tobacco dependence among khat users who also use tobacco, and specific tobacco cessation interventions should be developed to target this population group.

## Figures and Tables

**Table 1:** Details of included studies.

Study ID	Methodology	Methodological quality	Population/setting	Prevalence results	Additional results
Al-Dubai and Rampal, 2012 [40]	(i) Sampling frame: four main government hospitals (*N* = not reported) (ii) Sampling method: universal sampling (all doctors) (iii) Recruitment method: in person (iv) Administration method: in person, self-administered	(i) Sample size calculation: no (ii) Sampling type: nonprobability sampling (broad sampling, covered all doctors) (iii) Validity of tool: for khat/tobacco: self-developed tool, no validation reported (iv) Pilot testing done: no (v) Response rate: 70.4%	(i) Population: Yemeni doctors (ii) Country: Yemen (iii) Setting: hospitals (iv) *n* (sampled) = 800 (v) *n* (participated) = 563 (vi) *n* (included in analysis) = 563	(i) Prevalence of khat, *n* (%): 248 (44.0%) (sometimes 21.7%, frequently 9.95%, and daily 12%) (ii) Prevalence of tobacco use (specify tobacco type and pattern of use), *n* (%): 99 (17.6%) (cigarettes, pattern not specified) (iii) Prevalence of tobacco among khat chewers (specify tobacco type and pattern of use), cigarette, *n* (%): 84/248 (33.9%)	(i) Level of dependence among khat chewers who use tobacco: not reported (ii) Associated factors: cigarette smoking among khat chewers is significantly associated with male gender (AOR 3.77 (1.10–12.92)), working in government and private compared to government only (0.40 (0.21–0.75)), and for working > 10 years compared to less than 10 years (AOR 3.54 (1.44–8.67)). Model also adjusted for age, marital status, residence, and income

Alem et al., 1999 [41]	(i) Sampling frame: nine peasant associations and one urban dwellers association (*N* = 15,000) (ii) Sampling method: cluster random sampling (iii) Recruitment method: in person (iv) Administration method: in person, self-administered (with assistance)	(i) Sample size calculation: no (ii) Sampling type: probability (iii) Validity of tool: self-developed tool, validation reported (questions rephrased after showing them to pilot group of forty adults) (iv) Pilot testing done: yes (v) Response rate: 85.1%	(i) Population: persons aged over 15 years resident in Butaijra town (ii) Country: Ethiopia (iii) Setting: house-house (community-based) (iv) *n* (sampled) = 12,531 (v) *n* (participated) = 10,658 (vi) *n* (included in analysis) = 10,468	(i) Prevalence of khat, *n* (%): 911 (8.7%) daily, 5234 (50%) current (pattern not specified) (ii) Prevalence of tobacco use (specify tobacco type), *n* (%): 186 (1.8%) were mild cigarette smokers, 132 (1.3%) were moderate cigarette smokers, and 141 (1.3%) were heavy cigarette smokers (iii) Prevalence of tobacco among khat chewers (specific tobacco type), *n* (%): among daily khat chewers, 46 (5.0%) were mild (1–3 daily) cigarette smokers, 47 (5.2%) were moderate (4–9 daily) cigarette smokers, and 59 (6.5%) were heavy (>9 daily) cigarette smokers (iv) Pattern of tobacco use among khat chewers: we only have data for daily cigarette smokers (v) Daily khat and tobacco (cigarette): 46 + 47 + 59 = 152 *∗* 100/911 = 17%	(i) Level of dependence among khat chewers who use tobacco: not reported (ii) Associated factors: not reported

Al-Sanosy, 2009 [21]	(i) Sampling frame: total number of students enrolled in 11 colleges in the region (*N* = 18243) and secondary schools (*N* = 46760), total 102 schools (ii) Sampling method: systematic random sampling (iii) Recruitment method: in person (iv) Administration method: in person, self-administered	(i) Sample size calculation: yes (ii) Sampling type: probability sampling (iii) Validity of tool: previously reported validated tool (iv) Pilot testing done: yes (v) Response rate: 89.7%	(i) Population: secondary school and college students, 51.7% male, mean age 18.9 (2.58) years (ii) Country: Saudi Arabia (iii) Setting: secondary schools and colleges, May 2006 (iv) *n* (sampled) = 10,000 (v) *n* (participated) = 8965 (vi) *n* (included in analysis) = 8965	(i) Prevalence of khat, *n* (%) (specify pattern of use): past 30-day use: 1795/8965 (20.0%), daily khat chewers: 250/8965 (2.8%), most of week days: 322/8965 (3.6%), weekends: 765/8965 (8.5%), and occasionally: 468/8965 (5.2%) (ii) Prevalence of tobacco use (specify tobacco type and pattern of use), *n* (%): smoking (not defined) 863/8965 (9.6%) (iii) Prevalence of tobacco among khat chewers (specific tobacco type and pattern of use), *n* (%): 863/1101 (78.4%) of khat chewers were also cigarette smokers (iv) Mentioned only cigarettes	(i) Level of dependence among khat chewers who use tobacco: Not reported (ii) Associated factors: not reported

Alsanosy et al., 2013 [15]	(i) Sampling frame: intermediate educational level and high school students in the Jazan region (ii) Sampling method: cluster multistage sampling (iii) Recruitment method: in person (iv) Administration method: in person, self-administered	(i) Sample size calculation: yes (ii) Sampling type: probability sampling (iii) Validity of tool: previously reported validated tool (iv) Pilot testing done: yes (v) Response rate: 95.68%	(i) Population: full-time intermediate and high school students aged 13–21 years (75.1% aged 15–19 years, 56.3% male, and 61.3% urban residence) (ii) Country: Saudi Arabia (iii) Setting: schools in academic year 2011/2012 (iv) *n* (sampled) = 4100 (v) *n* (participated) = 3923 (vi) *n* (included in analysis) = 3923	(i) Prevalence of khat, *n* (%): current (past 30 days): 806/3923 (20.5%, 95% CI 19.27–21.79); ever: 952/3923 (24.2%, 95% CI 22.9–25.57) (ii) Prevalence of tobacco use (specify tobacco type), *n* (%): smoking status yes/no: 627 (17.3%) (iii) Prevalence of tobacco among khat chewers (specific tobacco type), *n* (%): cigarettes 489 (54.3%).	(i) Level of dependence among khat chewers who use tobacco: not reported (ii) Associated factors: not reported

Ayana and Mekonen, 2004 [42]	(i) Sampling frame: all registered university students in 2001/2002 (*N* = 2073) (ii) Sampling method: systematic random sampling (iii) Recruitment method: not reported (iv) Administration method: in person self-administered	(i) Sample size calculation: yes (ii) Sampling type: probability sampling (iii) Validity of tool: previously reported validated tool (iv) Pilot testing done: yes (v) Response rate: 94.4%	(i) Population: university students, mean age 24 (range 16–46), 76.91% male, 59.5% orthodox, and 49.2% Amhara ethnicity (ii) Country: Ethiopia (iii) Setting: university, January 2002 (iv) *n* (sampled) = 500 (v) *n* (participated) = 472 (vi) *n* (included in analysis) = 472	(i) Prevalence of khat, *n* (%) (specify pattern of use): current (daily, weekly, or occasionally) prevalence 117/472 (24.79%), divided into 52/472 (11.0%) every day, 35/472 (7.4%) once a week, and 30/472 (6.4%) occasionally (ii) Prevalence of tobacco use (specify tobacco type and pattern of use), *n* (%): 64/472 (13.56%) were cigarette smokers (pattern not defined) (iii) Prevalence of tobacco among khat chewers (specific tobacco type and pattern of use), *n* (%): among khat chewers, 53/117 (45.3%) were cigarette smokers. 16/117 (13.7%) of students smoke cigarettes while chewing khat; 8/117 (6.8%) of students smoke cigarettes after chewing khat	(i) Level of dependence among khat chewers who use tobacco: not reported (ii) Associated factors: not reported

Gorsky et al., 2004 [43]	(i) Sampling frame: one city in Israel (not mentioned) (ii) Sampling method: not reported (iii) Recruitment method: not reported (iv) Administration method: not reported	(i) Sample size calculation: no (ii) Sampling type: not reported (iii) Validity of tool: not reported (iv) Pilot testing done: no (v) Response rate: not reported	(i) Population: Yemenite Jews (all > 30 years old, male, and parents born in Yemen) (ii) Country: Israel (iii) Setting: unnamed city (iv) *n* (sampled) = 1500 (v) *n* (participated) = 1500 (vi) *n* (included in analysis) = 47	(i) Prevalence of khat, *n* (%) (specify pattern of use): 102/1500 (6.8%) (all used khat > 3 years) (ii) Prevalence of tobacco use (specify tobacco type and pattern of use), *n* (%): N/A (iii) Prevalence of tobacco among khat chewers (specific tobacco type and pattern of use), *n* (%): 32/47 (68%) were cigarette smokers and smoked more (29.5/day) than nonchewers (22.3/day) *p* = 0.03	(i) Level of dependence among khat chewers who use tobacco: not reported (ii) Associated factors: not reported

Kebede, 2002 [44]	(i) Sampling frame: all university instructors in four northwestern colleges (*N* = 321) (ii) Sampling method: universal sampling (iii) Recruitment method: not reported (iv) Administration method: self-administered	(i) Sample size calculation: no (ii) Sampling type: nonprobability (broad sampling, covered all instructors in four colleges) (iii) Validity of tool: not reported (iv) Pilot testing done: yes (v) Response rate: 75.1%	(i) Population: university instructors, 93.9% male, mean age 35.2 (8.36) years, 73.5% Christian, 12.7% Protestant, and 91.7% Ethiopians (ii) Country: Ethiopia (iii) Setting: colleges, January 2001 (iv) *n* (sampled) = 241 (v) *n* (participated) = 181 (vi) *n* (included in analysis) = 181	(i) Prevalence of khat, *n* (%) (specify pattern of use): lifetime khat use 59/181 (32.6%), current (past 30 days) use 38/181 (21.0%) (ii) Prevalence of tobacco use (specify tobacco type and pattern of use), *n* (%): lifetime cigarette use 51/181 (28.2%), current (past 30 days) use 24/181 (13.3%) (iii) Prevalence of tobacco among khat chewers (specific tobacco type and pattern of use), *n* (%): 34/59 (57.6%) of lifetime khat chewers were lifetime cigarette smokers, and 14/38 (36.8%) of current khat chewers were current cigarette smokers	(i) Level of dependence among khat chewers who use tobacco: not reported (ii) Associated factors: not reported

Lemma et al., 2012 [14]	(i) Sampling frame: all students enrolled at two universities (*N* = 29823) (ii) Sampling method: multistage sampling (iii) Recruitment method: not reported (iv) Administration method: self-administered	(i) Sample size calculation: no (ii) Sampling type: probability sampling (iii) Validity of tool: not reported (iv) Pilot testing done: no (v) Response rate: not reported	(i) Population: students, mean age 21.6 (1.7) years, and 77.3% male (ii) Country: Ethiopia (iii) Setting: universities (iv) *n* (sampled) = not reported (v) *n* (participated) = 2817 (vi) *n* (included in analysis) = 2230	(i) Prevalence of khat, *n* (%) (specify pattern of use): 209/1960 (10.7%) (1-2 and ≥ 3 times per week) (ii) Prevalence of tobacco use (specify tobacco type and pattern of use), *n* (%): 84/2230 (3.8%) ever cigarette smokers (iii) Prevalence of tobacco among khat chewers (specific tobacco type and pattern of use), *n* (%): 28.7% of khat chewers were current cigarette smokers, 2.4% of khat chewers were former cigarette smokers	(i) Level of dependence among khat chewers who use tobacco: not reported (ii) Associated factors: not reported

Reda et al., 2012 [36]	(i) Sampling frame: all students enrolled in high schools in Harar town (capital city of one of the nine regions of Ethiopia) (*N* = 6523) (ii) Sampling method: stratified random sampling (iii) Recruitment method: not reported (iv) Administration method: Self-administered	(i) Sample size calculation: no (ii) Sampling type: probability sampling (iii) Validity of tool: not reported (iv) Pilot testing done: yes (v) Response rate: 91.1%	(i) Population: high school students, 50.1% male, mean age 16.4 (1.6) years, 48.9% 9th grade, 52.8% Orthodox, 32.2% Catholic, and 11.2% Muslim (ii) Country: Ethiopia (iii) Setting: high schools, April 2010 (iv) *n* (sampled) = 1890 (v) *n* (participated) = 1721 (vi) *n* (included in analysis) = 1721	(i) Prevalence of khat, *n* (%) (specify pattern of use): 427 (24.2% 95% CI 22.2–26.2) had ever chewed khat, 89/1721 (5.2%) chewed khat daily (ii) Prevalence of tobacco use (specify tobacco type and pattern of use), *n* (%): 4.2% were current (past 30 days) smokers, and 12.4% were ever smokers (iii) Prevalence of tobacco among khat chewers (specific tobacco type and pattern of use), *n* (%): among ever chewers, 128/427 (29.9%) used waterpipe when they chewed khat	(i) Level of dependence among khat chewers who use tobacco: not reported (ii) Associated factors: not reported
